# Biomedical Application of Dental Tissue-Derived Induced Pluripotent Stem Cells

**DOI:** 10.1155/2016/9762465

**Published:** 2016-02-17

**Authors:** Jung-Hwan Lee, Seog-Jin Seo

**Affiliations:** ^1^Institute of Tissue Regeneration Engineering (ITREN), Dankook University, Cheonan 330-714, Republic of Korea; ^2^Department of Nanobiomedical Science and BK21 PLUS NBM Global Research Center for Regenerative Medicine, Dankook University, Cheonan 330-714, Republic of Korea

## Abstract

The academic researches and clinical applications in recent years found interest in induced pluripotent stem cells (iPSCs-) based regenerative medicine due to their pluripotency able to differentiate into any cell types in the body without using embryo. However, it is limited in generating iPSCs from adult somatic cells and use of these cells due to the low stem cell potency and donor site morbidity. In biomedical applications, particularly, dental tissue-derived iPSCs have been getting attention as a type of alternative sources for regenerating damaged tissues due to high potential of stem cell characteristics, easy accessibility and attainment, and their ectomesenchymal origin, which allow them to have potential for nerve, vessel, and dental tissue regeneration. This paper will cover the overview of dental tissue-derived iPSCs and their application with their advantages and drawbacks.

## 1. Introduction

Induced pluripotent stem cells (iPSCs) are a type of pluripotent stem cells that can be generated directly from adult somatic cells. In 2006, iPSCs with properties similar to embryonic stem cells (ESCs) could be generated from mouse fibroblasts by simultaneously transducing four exogenous OSKM genes (Oct3/4, Sox2, Klf4, and c-MYC) [1]. In 2007, human iPSCs were generated by human fibroblasts using the same way [2]. On the same day, James Thomson's group also reported the generation of human iPSC using a different combination of factors (Oct4, Sox2, Nanog, and Lin28) [3]. Then, Shinya Yamanaka was awarded the 2012 Nobel Prize along with Sir John Gurdon “for the discovery that mature cells can be reprogrammed to become pluripotent.” [4]. Since then, iPSCs have held great promise in the field of regenerative medicine due to their pluripotency able to differentiate into any cell types in the body (such as neurons, heart, pancreatic, and liver cells) without using embryo, which could be used to replace damaged or diseased tissues or organs [5]. Before introduction of iPSCs, the most well-used pluripotent stem cell was ESCs. However, embryo could be destructed for the use of ESCs, and this may cause many ethical and legal concerns. Therefore, iPSCs have emerged as a new tool in the practical application for regenerative medicine, such as the treatment of diabetes mellitus platelet deficiency, Parkinson's disease, platelet deficiency, macular degeneration and spinal cord injury [6–10].

**Figure 1:**
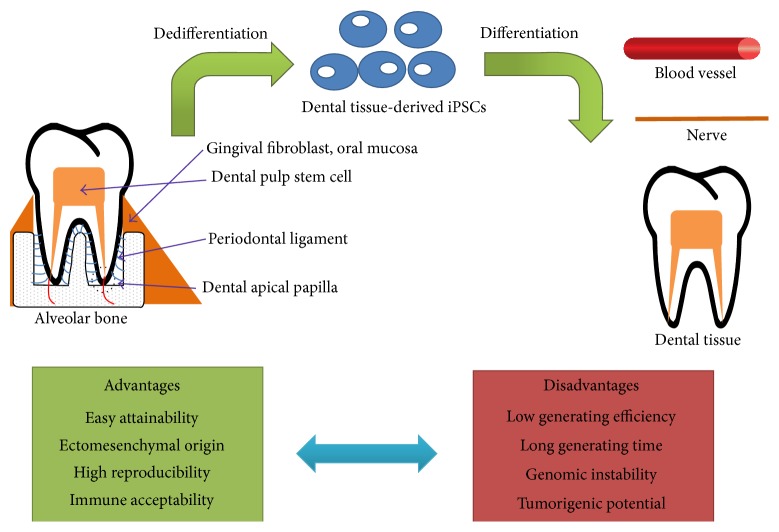
Schematic image showing sources of iPSCs in dental tissue, their regenerative application, advantages and disadvantages.

Despite their promise, iPSCs generation from human fibroblast is limited in the clinical application due to lack of accessibility [11]. Patients are afraid of the invasive ways such as living tissue cutting for getting donor cells and generating iPSCs. Compared to skin fibroblast and other types of cells, cells from dental tissue are able to be easily attainable in minimally invasive way. Researchers can use oral mucosa or gingival tissue, which are easily obtained with wiping target tissue with cotton swab in oral cavity, and extracted third molar or deciduous teeth which have been treated as biomedical wastes [12]. In addition, dental tissue from dental pulp, periodontal ligament, and apical papilla have abundant stem cells, which are readily dedifferentiated into iPSCs than other somatic fibroblasts [13]. Therefore, along with the easy attainability and possibility of incorporating stem cells in dental tissue, iPSC generation from dental tissues has been widely investigated and dental tissue-derived iPSCs are considered as a type of promising regenerative precursors for nerve, blood vessel, and dental tissue regeneration due to their ectomesenchymal origin [14, 15]. Sources of iPSCs in dental tissue and regenerative application were summarized with their advantages and disadvantages (Figure 1).

In addition, iPSCs possess a potential for treating such genetically oriented disorders using availability of disease-specific iPSCs from the patient, which are available to investigate disease-specific treatments [13]. Although most of the genetic-related disease studies are undertaken using knock out mouse models, genetic disorders or defects found in human may not induce the same symptoms in mouse. Therefore, cell cultures from human diseased tissues are considered to be the most suitable complement to human trial study and animal models. Growing evidence illustrates that disease-specific iPSCs are placed in a niche for patient-specific therapy with their genomic match with patient and similarity of disease state [11]. In addition, genetic disorders and chronic degenerative dental diseases such as dental caries and periodontitis are widespread in human populations and represent a significant problem for public health. To repair dental tissues damaged from the above chronic degenerative dental diseases, regeneration of enamel, dentin, dental pulp, periodontal ligament, alveolar bone, and their complex would be performed using dental tissue-derived iPSCs due to their pluripotency and epigenetic memory [16]. Therefore, the use of dental tissue-derived iPSCs could be a promising therapeutic tool in biomedical regeneration for treating genetically oriented systemic diseases or chronic degenerative dental diseases.

## 2. History of Dental Tissue-Derived iPSCs

iPSC technology was established on the basis of numerous findings. There were three major streams of research that led researchers to the production of iPSCs. The first stream was reprogramming by nuclear transfer by John Gurdon in 1962, which reported success of generating tadpoles from unfertilized eggs that had received a nucleus from the intestinal epithelium cells of adult frogs [17]. More than three decades later, Ian Wilmut and colleagues reported the first cloned mammal generated by transferring a single nucleus to an enucleated unfertilized egg, which demonstrated that even differentiated adult cells contain all of the genetic information required for the development of entire organisms and that oocytes have potential of reprograming somatic cell nuclei [18]. In the 21st century, Takashi Tada's group showed that fusion of adult cells with ESCs gave a potential of somatic nuclear reprogramming [19].

The second stream was related to the discovery of a “master” transcription factor. Many researchers began to search for a “master” regulator to determine cell fate or reprogram various cell lineages. In 1987, a* Drosophila* transcription factor (Antennapedia) was revealed to induce the formation of legs instead of antennae when ectopically expressed [20] and a mammalian transcription factor (MyoD) was shown to convert embryonic fibroblasts into myoblasts [21]. These results led to the concept of a “master” transcription factor that determines or induces and reprograms the fate of a given cell lineage.

The third stream is about ESCs. Since the first generation of mouse ESCs [22], researchers have established culture conditions that enable the long-term maintenance of mouse ESCs's pluripotency with leukemia inhibitory factor (LIF) [23]. Likewise, since the first generation of human ESCs, optimal culture conditions have been established with additive such as basic fibroblast growth factor (bFGF) [24].

Combining the first two streams of research led Yamanaka's group to hypothesize that a combination of multiple factors in oocytes or ESCs was needed to reprogram somatic cells back into the embryonic state and they designed experiments to identify that combination of the genes among 24 candidates genes and found combination of four master factors which are determined as OSKM genes. With information about the culture conditions that are needed to culture pluripotent cells, researchers have been then able to identify generated iPSCs.

First success of inducing iPSCs used retroviral infection method to efficiently transport OSKM genes inside fibroblast [1]. Four years later, dental tissue-derived iPSCs were successfully established from stem cells from exfoliated deciduous teeth, stem cells from apical papilla, and DPSCs using OSKM or other 4 factors (Lin28, Nanog, Oct4, and Sox2) [11]. To date, iPSCs have been typically generated using either retroviruses or lentiviruses, which might cause insertional mutagenesis or the body to develop an immune response against the viruses. Thus, a viral infection transduction method would pose a risk for clinical application even though mice derived from retrovirally derived iPSCs are apparently normal as long as repression of c-Myc transgene is performed [25, 26]. Thus, for the purpose of cell transplantation therapy as clinical application, induction methods involving virus vector integration into the host genome should be avoided.

Many trials to generate viral-transgene-free iPSCs have been performed. These methods using cDNA included plasmid, synthesized modified mRNAs,* Sendai virus* (SeV) vectors replicating the form of negative-sense single-stranded RNA in the cytoplasm of infected cells, double-stranded microRNAs (miRNAs), PiggyBac transposition, and cell penetrating peptide fused proteins have been suggested as viral-integration-free iPSCs generation method [27–32]. Among these, plasmids and* Sendai virus* are now routinely used in many laboratories due to their simplicity and reproducibility but there are still hurdle regarding low efficiency of the process, which showed less efficiency than typical iPSCs generating process which is transfecting fibroblasts with retroviruses or lentiviruses [5]. Therefore, scientists are now shifting their efforts from biological technology development to material technology development for increasing efficiency in nonviral system. Magnetic nanoparticles, cationic bolaamphiphile, poly-*β*-amino esters, polyketal nanoparticle, calcium phosphate nanoparticle, and polyamidoamine nanoparticles based nonviral transfection have been performed for generating iPSCs with high efficiency [33–38]. External force such as electromagnetic fields (EMFs) is revealed to mediate efficiency of cell reprogramming into a pluripotent state by directly regulating dynamic epigenetic changes via the induction of Mll2, a histone lysine N-methyltransferase [39]. In addition, library studies using four different polymers and graphene modified substrate have shown increased efficiency of cell reprogramming [40, 41]. Unfortunately, most of attempts to establish nonviral iPSCs generating system with high efficiency have been performed in fibroblast-derived iPSCs. Further experiments regarding dental tissue-derived iPSCs with optimal nonviral system consisting of appropriate carrier, substrate materials and topographical characteristics, and external force will be needed for use in regenerative medicine.

## 3. Advances in Dental Tissue-Derived iPSCs

Dental tissue-derived adult stem cells, such as dental pulp stem cells, periodontal ligament stem cells, dental follicle stem cells, stem cells from apical papilla, and stem cells from human exfoliated deciduous teeth have lots of advantages. One of them might be their ease of isolation from extracted third molars or deciduous teeth. For this reason, they can be used for regenerative therapies, but their sources and populations are very limited. Alternatively, iPSCs have been highlighted as a next regenerative cell source since their cell sources are rich and they are very proliferative. Among many types of iPSCs depending on cell origin, dental tissue-derived iPSCs are able to be readily produced from easily assessable dental tissues. With the advance in cell extraction technology from tissue, iPSCs can be generated from readily available dental tissue sources, such as oral mucosa, gingival tissue, and dental tissue-derived stem cell, mentioned above [42, 43]. Among them, oral mucosa and gingival tissue are able to be easily obtained from oral cavity and saliva, which can be accessible to the original tissue compared to other dental tissue-derived stem cells extracted from teeth. More importantly, dental tissue-derived iPSCs are more proliferative than dental tissue-derived stem cell when they are cultured in vitro, which would be required for use in regenerative therapies in the dental or medical clinic.

As another advantage, dental tissue-derived iPSCs are reproducible compared to dental tissue-derived stem cells which are not able to guarantee the reproducibility of regenerative potency in clinic due to their limitedly attainable stem cell incorporated dental tissue. This reproducibility is able to be provided for clinical application of dental tissue-derived iPSCs in regenerative medicine.

The other advantage of dental tissue-derived iPSCs in regenerative medicine is to maintain epigenetic memory of the source tissue according to previously reported studies. The epigenetic memory induces preferential lineage-specific differentiation of iPSCs, which means that generated iPSCs will preferentially differentiate back to the original cell type [12, 44, 45]. Therefore, dental tissue-derived iPSCs could promote their capacity to differentiate into nerve, vessel, dental hard tissue, and other types of dental tissues. On the other hand, ESCs have less potency to differentiate into certain types of target tissues due to lack of epigenetic memory. However, when it comes to application in other regenerative medicine except nerve, vessel, and other types of dental tissues, dental related epigenetic memory turns out to be a major hurdle to get success.

The major advantage of dental tissue-derived iPSCs over ESCs cells is that dental tissue-derived iPSCs can be derived from a patient's own dental tissue, thereby preventing immune rejection after transplantation and the ethical concerns regarding the use of ESCs [46]. Additionally, dental tissue-derived iPSCs can be made for disease-specific stem cells that are able to carry donor's genome and mimic human diseases more reliably than other animal models [13]. Disease-specific iPSCs from individuals who have different disease state allow better understanding of the complexity and nature of a disease depending on the stage of disease [2]. Therefore, disease-specific iPSCs help researchers to find out disease-specific drugs and treatments. From now on, most of the genetic-related disease studies are undertaken using genetically modified rodent models, but these research results may be difficult to be applied to human-related genetic disorders or defects. Therefore, human tissue cell cultures are considered to be the best for human-related genetic disorders or diseases but they have limited proliferative potency. Therefore, iPSCs are considered as promising candidate of unique stem cell type to study human-related disorders or diseases and dental tissue-derived iPSCs are paid attention with advantage of easy accessibility. Until now, generating disease-specific iPSCs has been mostly studied using fibroblast for Parkinson's disease, Huntington's disease, Down syndrome, Juvenile diabetes mellitus, Shwachman–Bodian–Diamond syndrome, and so on [47]. Therefore, dental tissue-derived iPSCs from disease-specific specimen are expected to provide a promising platform for the investigation of disease mechanisms, drug discovery, and personalized treatments with advantage of easy accessibility.

## 4. Limitations of Dental Tissue-Derived iPSCs

The most arising problems using iPSCs in regenerative medicine may be safety concerns. Dental tissue-derived iPSCs cannot escape these issues. All iPSCs have genomic instability and a big rate of tumorigenicity in vivo [48, 49]. The use of viral integrating vectors for the generation of iPSCs may contribute to inducing genomic instability and tumorigenic potential of iPSCs. To reduce these, many researchers have attempted to utilize virus-free generation iPSCs [33, 35, 38]. These attempts to use viral vectors have provided a platform to develop the technology of nonviral vectors for potential medical or dental application. Furthermore, the tumorigenicity of iPSCs is able to be minimized by differentiating into mature cells or into lineage-specific progenitor cells such as MSCs prior to use in regenerative therapies [50]. Combining differentiation of iPSCs into lineage-specific progenitor cells such as MSCs and dental pulp stem cells and the use of nonviral systems in iPSCs generation will help to overcome the major safety issues currently associated with the use of iPSCs in the regenerative medicine.

Some issues about risks of infectious diseases and unwanted immunogenicity from animals have been raised regarding current iPSCs generating protocols. Current protocols use reagents of animal origin (mainly fetal bovine serum, FBS) that carry the potential risks of infectious diseases and unwanted immunogenicity, which is associated with a variety of quality control and safety issues [51]. Exact composition of bovine serum is unknown and varies from batch to batch, resulting in interfering with the reproducibility of iPSCs generation. Moreover, serum could be contaminated with viruses, mycoplasma, prions, or other pathogenic, toxic, or immunogenic agents [52]. Although little is known regarding other xenogeneic products, porcine-derived trypsin is likely to contain similar biosafety risks. Therefore, further research is needed to establish and investigate a concrete protocol to isolate and expand donor cells and iPSCs while guaranteeing clinical safety and efficiency of iPSCs generation by completely removing or replacing animal serum with chemically defined materials for cell-based regenerative medicine.

Although iPSCs can be generated simply and reproducibly, generating efficiency into iPSCs from donor cells remains very low (≈0.01%) using fibroblasts. Generation efficiency of iPSCs could be increased 4–10 times greater (≈0.1%) than fibroblasts when using dental derived tissues such as DPSCs, stem cells from shed primary teeth or extracted permanent teeth, and stem cells from human exfoliated deciduous teeth. But these generating efficiency from dental tissue is still low for scalable regenerative medicine. Along with the complicity of process and long generating time (≈2 weeks), low efficiency of dental tissue-derived iPSCs will induce spending much time in proliferating them, transferring into target site, and generating regenerative biomolecules from iPSCs cultured media, which may cause missing ideal treatment period for regeneration.

Despite drawbacks associated with dental tissue-derived iPSCs, the potential that iPSCs have demonstrated for regenerative medicine and genetic disorders demands further research to minimize these remedial hazards. Therefore, the establishment of a safe and efficient process for generating iPSCs is required for regenerative application along with a better understanding of the biology of cellular reprogramming.

## 5. Biomedical Application of Dental Derived iPSCs

Dental tissue-derived iPSCs are able to be generated by various somatic dental tissues or stem cells like DPSCs. DPSCs are retained in the soft living tissue inside the tooth, which is considered as a very special tissue. Dental pulp is a specific tissue originating from ectomesenchyme possessing similar properties of mesenchyme which is a precursor of mesodermal cells (blood, tubular, muscle, and so on) and neural crest cells and is enclosed into a dental cavity surrounded by mineralized dentin. DPSCs have been applied in biomedical regeneration due to their promising potential to differentiate into a variety of other cell types including myocardiocytes to repair damaged cardiac tissue, neuron to generate nerve and brain tissue, myocytes to repair muscle, osteocytes to generate bone, and chondrocytes to generate cartilage [53–56]. Owing to the great nature potential for various biomedical applications, DPSCs have been mainly chosen for a type of precursors for iPSCs among other dental tissues, and DPSC-derived iPSCs have been used for the regeneration of neuron, blood vessel, and teeth.

DPSC-derived iPSCs can successfully differentiate into neuroectodermal lineage neuron-like cells, which resemble neurons both morphologically and functionally and could be used for neuron regeneration [11, 46, 57]. Even though there were few reports establishing neural lineages from skin fibroblast-derived iPSCs for nerve tissue regeneration [58, 59], generation of neural lineages from DPSC-derived iPSCs has been repeatedly reported due to its mesodermal-like origin. Recently, establishment of the in vitro disease models using DPSC-derived iPSCs was carried out for a variety of neuropsychiatric disorders such as schizophrenia and autism spectrum disorders. After investigation of an extensive gene expression profiling analysis of differentiated neurons from DPSCs and skin fibroblast-derived iPSCs, DPSC-derived iPSCs were chosen for disease models of neuropsychiatric disorders because of their developmental origins [15]. Even though more investigation is needed to confirm genetic similarities between neural crest stem cells and generated neural lineage from DPSC-derived iPSCs compared to skin fibroblast-derived iPSCs, DPSC-derived iPSCs are considered as a type of promising precursors for neural regeneration and establishing disease modeling.

Bhattacharjee et al. reported the generation of DPSC-derived iPSCs using only two nononcogenic factors (Oct4 and Sox2) and their feasibility as substrates for endothelial progenitor cells (EPCs) [60]. Under conventional CD34^+^ EPCs differentiation conditions, DPSC-derived iPSCs showed higher efficiency in differentiation into functional endothelial and smooth muscle cells than normal iPSCs from fibroblasts. The angiogenic and neovasculogenic activities were confirmed in mouse models of hind-limb ischemia and myocardial infarction after transplantation, resulting in a promising strategy for patient-specific EPC therapies and disease modeling, particularly for ischemic vascular diseases. As a type of promising regenerative precursors, EPCs differentiated from iPSCs can be used as novel ingredients for the patient-specific EPC therapies [61–63]. Even though there have been abundant interests in EPCs therapies for vascular disease, few reports on EPCs from DPSC-derived iPSCs have been published.

In recent years, regenerative medicine has been focusing on a new strategy over dental tissue-derived stem cells to regenerate teeth for tooth replacement. With the rapid development of iPSCs technology, dental tissue-derived iPSCs can serve as a novel nonodontogenic stem cell source for a tissue-engineered tooth-like structure [64]. To explore the ability of iPSCs to differentiate into odontogenic cells, recombinant tooth germ model can be fabricated by mixing various types of dental cells such as mesenchymal cells, epithelial cells, dental tissue-derived iPSCs, and specifically differentiated iPSCs [65]. After the patient's somatic cells are harvested from dental tissue and generating patient-specific iPSCs, specifically iPSCs differentiated into ectodermal epithelial cells and neural crest-derived mesenchymal cells are combined by direct contact, mimicking the in vivo arrangement and enabling forming odontogenic cells. Although this could be suggested as a promising strategy, unfortunately, there was less reports on this concept.

## 6. Concluding Remarks

Despite substantial progress in researches on dental tissue-derived iPSCs, significant challenges must be addressed for reaching clinical application. It is evident that dental tissue-derived iPSCs could be of use in neuron, blood vessel, and teeth regeneration due to highly accessible attainment, reproducibility, capability of self-renewal and large-scale expansion, less immune rejection, avoidance of ethical controversy, and differentiation toward all the three germ layers, especially neuron and vessel. However, the generation of functional teeth using these cells will almost certainly remain elusive in the foreseeable future due to low efficiency in their generation and possibilities of risks of genomic instability, tumorigenicity, infectious diseases, and unwanted immunogenicity. To conclude, this review confirms a potential that dental tissue-derived iPSCs and their derivatives contribute to enhancing the regeneration of nerve, vessel, and dental tissues; however, further studies are required to evaluate efficacy and safety prior to human clinical trials.
